# Atropisomeric determination of chiral hydroxylated metabolites of polychlorinated biphenyls using HPLC-MS

**DOI:** 10.1186/1752-153X-7-183

**Published:** 2013-12-22

**Authors:** Guangshu Zhai, Xianai Wu, Hans-Joachim Lehmler, Jerald L Schnoor

**Affiliations:** 1 Department of Civil and Environmental Engineering and IIHR Hydroscience and Engineering, The University of Iowa, Iowa City 52242, IA, USA; 2 Department of Occupational and Environmental Health, The University of Iowa, Iowa City 52242, IA, USA

**Keywords:** Chiral polychlorinated biphenyls, Hydroxylated metabolites, HPLC-MS, Rat liver microsome

## Abstract

**Background:**

Polychlorinated biphenyls (PCBs) are a group of environmental persistent organic pollutants, which can be metabolized into a series of metabolites, including hydroxylated metabolites (OH-PCBs) in biota. Nineteen of 209 PCB congeners can form chiral stable isomers. However, atropisomeric determination of the hydroxylated metabolites of these chiral PCBs has never been reported by LC methods. In this work, a novel HPLC-MS method was developed to detect five chiral OH-PCBs (4OH-PCB91, 5OH-PCB91, 4OH-PCB95, 5OH-PCB95 and 5OH-PCB149) using HPLC-MS without a derivatization step.

**Results:**

The influences of column-type, column temperature, flow rate and ratio of the mobile phase on the atropisomeric separation were investigated in detail. In the final method, calibration curves, based on peak areas against concentration, were linear in a range of 1–100 ng mL^-1^ of five chiral OH-PCBs with correlation coefficients ranging from 0.9996 to 0.9999 for all atropisomers of OH-PCBs. The relative standard deviations measured at the 10.0 ng mL^-1^ level for atropisomers of five chiral OH-PCBs were in the range of 0.60-7.55% (n = 5). Calculated detection limits (S/N = 3) of five chiral OH-PCBs were between 0.31 and 0.60 ng mL^-1^ for all OH-PCB atropisomers.

**Conclusion:**

This HPLC-MS method was developed to detect chiral OH-PCBs and further successfully applied to measure OH-PCB atropisomer levels and enantiomeric fractions (EFs) in rat liver microsomal samples. The results from LC-MS method were highly consistent with those from GC-ECD method. It is the first time to report these OH-PCB atropisomers detected in microsomes by HPLC-MS. The proposed method might be applied also to detect chiral OH-PCBs in environmental samples and for metabolites of PCBs *in vivo*.

## Background

The persistence and wide application of polychlorinated biphenyls (PCBs) in past decades have led to their ubiquitous occurrence in the environment [1]. PCBs can adversely affect human health via bioaccumulation and biomagnification in the food chain [2]. Among 209 congeners, nineteen of PCBs with 4–8 chlorines are chiral and they can form stable rotational isomers, or atropisomers, that are nonsuperimposable mirror images of each other [3]. Furthermore, at least 12 chiral PCBs, including PCBs 91, 95 and 149, are present in commercial PCB mixtures [4]. Therefore, several chiral PCBs were detected in environmental media, such as soil [5,6], sediment [7], aquatic and riparian biota (fish, bivalves, crayfish, water snakes, barn swallows) [8], birds [9], shark and grouper [10], dolphins [11], whale [12], human feces and liver [13,14].

Chiral PCBs, like other chiral compounds, have special functions on elucidating the environmental and toxicological processes via their specific changes in enantiomeric fraction (EF) (enantioselectivity) during their metabolism and degradation because only the biological factors can affect the EF changes. The latest comprehensive review on chiral PCBs transport, metabolism and distribution covered the latest findings of chiral PCBs, including chiral PCB metabolites (such as methylsulfonyl-PCBs and hydroxy-PCBs), in the environmental and toxicological fields [15]. It was seen that the introduction of a hydroxy, methoxy or methylsulfonyl group will add an additional element of asymmetry so that hydroxy, methoxy or methylsulfonyl-PCBs are still chiral compounds [16]. Among the metabolites of chiral PCBs, hydroxy-PCBs (OH-PCBs) are frequently reported. It is well-known that the atropisomers of chiral PCBs showed different toxicity [15]. However, the field on toxicity of chiral metabolites of PCBs is still in its infancy. Therefore, it is necessary to develop sensitive and selective detection methods for chiral metabolites of PCBs, including chiral OH-PCBs.

Gas chromatographic methods with different detectors, such as mass spectrometry (GC-MS) [17-20] and electron capture detector (GC-ECD) [21-25], are presently the most common methods for the determination of OH-PCBs in environmental matrices. However, the interaction of the hydroxyl groups in OH-PCBs with basic sites in the injector and column leads to difficulties in direct analyses of OH-PCBs by gas chromatography. Abraham et al. [26] tried to directly detect the OH-PCBs but failed due to tailing chromatographic peaks and irreproducible peak areas. Derivatization of the OH-PCBs can eliminate the influence of the hydroxyl group on the separation and increase the volatility and stability, making them more amenable to gas chromatographic analysis. Thus, OH-PCBs can be derivatized using different derivatization reagents, such as diazomethane, diazoethane, acetyl, trifluoroacetyl, or pentafluoropropionyl analogues before gas chromatographic analysis [26-28]. However, the derivatization procedure is a time-consuming step and one of the main sources of error due to incomplete derivatization [27,29]. The separation of chiral OH-PCBs poses additional challenges because one chiral PCB congener may be metabolized into several chiral OH-PCBs, such as PCB95 producing 4OH-PCB95, 5OH-PCB95, and each chiral OH-PCB has two atropisomers. Up to now, chiral OH-PCBs, including OH-PCB 91, 95, 132, 136 and 149, have been successfully detected only by gas chromatographic methods [30-32].

High performance liquid chromatography-mass spectrometry (HPLC-MS) has the great advantage to determine chiral OH-PCBs because no derivatization step is needed, which avoids the loss of analyte during the derivatization step and saves a lot of time. However, there are more challenges to separate the atropisomers of chiral OH-PCBs for HPLC method than GC method because the resolution of HPLC shorter chiral columns is relatively lower than that of longer GC column. Therefore, few papers have reported the analysis of chiral PCB hydroxyl metabolites by HPLC-MS. Pham-Tuan et. al [33] reported the detection of 4-methoxy-2,2′,3,4′,5′,6 hexachlorobiphenyls (4MeO-PCB149), 3-methylsulfonyl-PCB149 (3MeSO_2_-PCB149), 4-methylsulfonyl-PCB149 (4MeSO_2_-PCB149) and 4-methylthionyl-PCB149 (4MeS-PCB149) using semipreparative HPLC on β-cyclodextrin-based columns. However, they found that none of the seven chiral columns evaluated in their study could separate the 4OH-PCB149 atropisomers under reversed-phase chromatographic conditions. Only the short Nucleodex β-OH, a native β-cyclodextrin column, gave a hint of an enantioselective separation.

Up to now, there are no successful reports of atropisomeric detection of chiral OH-PCBs by HPLC-MS. In this work, a HPLC-MS method to analyze the atropisomers of five chiral OH-PCBs (4OH-PCB91, 5OH-PCB91, 4OH-PCB95, 5OH-PCB95 and 5OH-PCB149) was developed and the critical parameters were optimized. The method was finally applied to determine the enantiomeric fractions (EFs) of chiral OH-PCBs obtained from incubations of chiral PCBs with rat liver microsomes prepared from phenobarbital (PB)-pretreated male rats. These results of chiral OH-PCBs from LC method were in agreement with an established GC-ECD method.

## Results and discussion

### Effect of column

Properties of the chiral column have a great influence on the separation of these chiral OH-PCBs. Four different chiral columns, including Nucleodex β-PM, Nucleodex β-OH, Astec Chirobiotic V and LichroCART 250–4 ChiraDex, were used to separate the atropisomers of five chiral OH-PCBs in this work. Nucleodex β-OH column was reported to give a hint of an enantioselective separation of 4OH-PCB149 [33], but it never showed any separation of atropisomers of OH-PCB91, OH-PCB95 and 5OH-PCB149, which suggested that the same chiral column has different enantioselective separation ability for different chiral OH-PCBs. Among the columns studied, only Nucleodex β-PM column could (partially) separate the atropisomers of 4OH-PCB91, 5OH-PCB91, 4OH-PCB95, 5OH-PCB95 and 5OH-PCB149 at different operating conditions of the LC-MS. Therefore, Nucleodex β-PM column was selected for all subsequent experiments.

### Effect of column temperature

**Figure 1:**
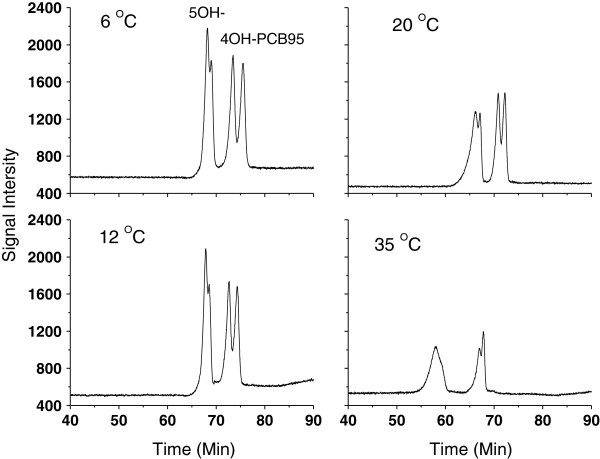
**Effect of column temperature on the atropisomeric separation of 10 ng mL**^**-1 **^**of mixture of 4OH-PCB95 and 5OH-PCB95.** Column: Nucleodex β-PM; Injection: 20 μL; Mobile Phase: A (water), B (acetonitrile); Gradient: 0 min (0.3 mL/min, A:B = 55:45) → 25 min (0.3 mL/min, A:B = 55:45) → 26 min (0.2 mL/min, A:B = 50:50) → 55 min (0.2 mL/min, A:B = 50:50) → 56 min (0.2 mL/min, A:B = 45:55) → 65 min (0.2 mL/min, A:B = 45:55) → 66 min (0.35 mL/min, A:B = 45:55) → 89 min (0.35 mL/min, A:B = 45:55) → 90 min (0.35 mL/min, A:B = 55:45) → Total 100 min.

The effect of column temperature on the separation of chiral OH-PCBs was studied between 6°C to 35°C using 4OH-PCB95 and 5OH-PCB95 as model compounds. While further lowering the column temperature may have improved the atropisomeric separation, temperatures below 6°C were considered impractical and not investigated. It can be seen from Figure 1 that the atropisomeric separation performance of 4OH-PCB95 and 5OH-PCB95 decreased sharply following the increase of column temperature from 6°C to 35°C. Two peaks of 5OH-PCB95 were merged into one peak and the atropisomers of 4OH-PCB95 can be slightly separated at 35°C. Therefore, the column temperature was set at 6°C in the subsequent parameter development process. Likewise, Pham-Tuan et al. [33] also found that the enantioselective separation quality of 4MeO-PCB149 on Nucleodex β-PM column improved significantly when the column temperature was set at a lower temperature than 35°C.

### Effect of flow rate and ratio of mobile phase

**Figure 2:**
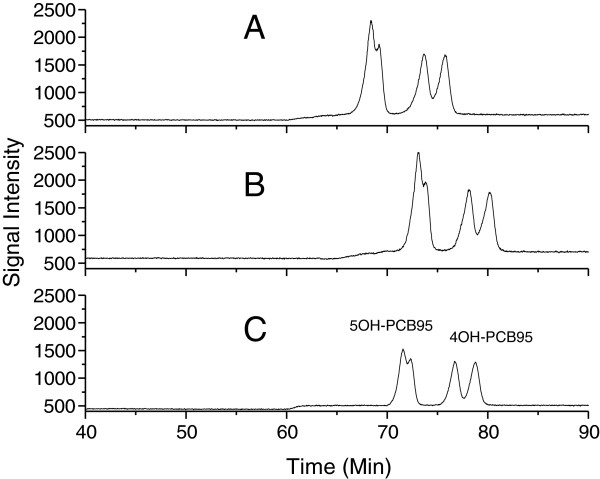
**Effect of mobile phase on the atropisomeric separation of 10 ng mL**^**-1 **^**of mixture of 4OH-PCB95 and 5OH-PCB95 at 6°C.** Column: Nucleodex β-PM; Injection: 20 μL; Mobile Phase: water and acetonitrile; mobile phase gradient (Table 1). **(A)**, chromatogram of condition I in Table 1; **(B)**, chromatogram of condition II in Table 1; **(C)**, chromatogram of condition III in Table 1.

**Figure 3:**
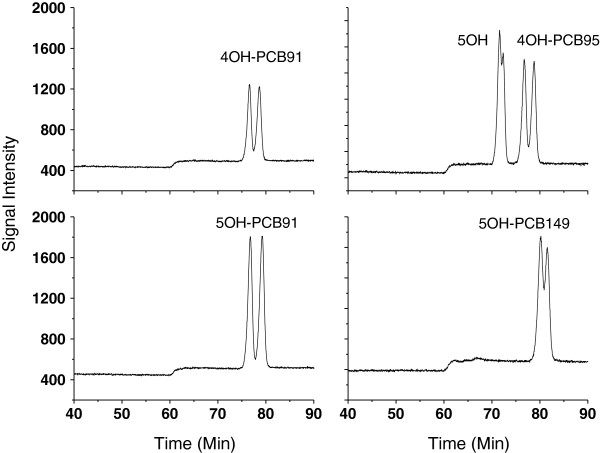
**Chromatograms of OH-PCBs (10 ng mL**^**-1 **^**of 4OH-PCB91; 10 ng mL**^**-1 **^**of 5OH-PCB91; 10 ng mL**^**-1 **^**of mixture of 4OH-PCB95 and 5OH-PCB95, 10 ng mL**^**-1 **^**of 5OH-PCB149) at Condition III for mobile phase in Table**1**.**

The composition of the mobile phase and its ratio are very important for the atropisomeric separation of OH-PCBs. Water and acetonitrile were selected as the mobile phase in this work. However, only a narrow ratio range (50-60% of acetonitrile) was confirmed to separate the OH-PCB atropisomers with much testing. These chiral OH-PCBs were hard to elute when the ratio of acetonitrile was below 50% and they eluted together when ratio of acetonitrile was above 60%. In addition, it was found that a gradient mobile phase can separate them better than isocratic conditions. Therefore, gradient mobile phase conditions were optimized in the following experiment. It can be seen from Figure 2 that condition III in Table 1 had the best separation for 4OH-PCB95 and 5OH-PCB95. Under this condition III, other OH-PCB atropisomers also displayed good separation as shown in Figure 3.

**Table 1:** **Time table of gradient mobile phases in Figure **2**: A (water), B (acetonitrile)**

**Condition**	**Time table of gradient mobile phases**
I	0 min (0.3 mL/min, A:B = 55:45) → 25 min (0.3 mL/min, A:B = 55:45) → 26 min (0.2 mL/min, A:B = 50:50) → 55 min (0.2 mL/min, A:B = 50:50) → 56 min (0.2 mL/min, A:B = 45:55) → 65 min (0.2 mL/min, A:B = 45:55) → 66 min (0.35 mL/min, A:B = 45:55) → 89 min (0.35 mL/min, A:B = 45:55) → 90 min (0.35 mL/min, A:B = 55:45) → Total 100 min
II	0 min (0.2 mL/min, A:B = 55:45) → 25 min (0.2 mL/min, A:B = 55:45) → 26 min (0.2 mL/min, A:B = 50:50) → 55 min (0.2 mL/min, A:B = 50:50) → 56 min (0.2 mL/min, A:B = 45:55) → 65 min (0.2 mL/min, A:B = 45:55) → 66 min (0.35 mL/min, A:B = 45:55) → 89 min (0.35 mL/min, A:B = 45:55) → 90 min (0.35 mL/min, A:B = 55:45) → Total 100 min
III	0 min (0.2 mL/min, A:B = 55:45) → 25 min (0.2 mL/min, A:B = 55:45) → 26 min (0.2 mL/min, A:B = 50:50) → 55 min (0.2 mL/min, A:B = 50:50) → 56 min (0.2 mL/min, A:B = 45:55) → 60 min (0.2 mL/min, A:B = 45:55) → 61 min (0.35 mL/min, A:B = 45:55) → 89 min (0.35 mL/min, A:B = 45:55) → 90 min (0.35 mL/min, A:B = 55:45) → Total 100 min

### Analytical performance

The analytical performance data of this optimized HPLC-MS method for the atropisomers of five chiral OH-PCBs are shown in Table 2. Linear calibration curves, based on peak areas to concentration, were obtained in the range of 1.0–100.0 ng mL^-1^, with correlation coefficients ranging from 0.9996 to 0.9999 for all OH-PCB atropisomers. The relative standard deviations measured at the 10.0 ng mL^-1^ level for OH-PCB atropisomers were in the range of 0.60-7.55% (n = 5). Calculated detection limits (*S/N* = 3) of five chiral OH-PCBs were between 0.31 and 0.60 ng mL^-1^ for all OH-PCB atropisomers. Thus, the method developed in this work had a relatively high sensitivity and low detection limit. The detection limits of the OH-PCBs in the GC-ECD method were considerably higher and ranged from 19.0 to 27.6 ng mL^-1^.

**Table 2:** The analytical performance data of the proposed LC-MS method

**Compound**	**Calibration curve**	**Correlation coefficient**	**Detection limit**	**RSD**
			**(ng mL**^**-1**^**)**	**(%, n = 5)**^**a**^
E1-5OH-PCB91	Y = 8697.4×-2090.9	0.9998	0.30	1.31
E2-5OH-PCB91	Y = 9261.9×-2124.1	0.9997	0.31	0.60
E1-4OH-PCB91	Y = 4831.3×-1493.0	0.9998	0.54	0.67
E2-4OH-PCB91	Y = 5009.9×-1607.8	0.9998	0.60	1.14
E1-5OH-PCB95	Y = 6172.1×-3904.2	0.9997	0.44	7.55
E2-5OH-PCB95	Y = 3822.2×-2213.2	0.9996	0.56	6.87
E1-4OH-PCB95	Y = 5275.7×-1726.8	0.9998	0.49	3.12
E2-4OH-PCB95	Y = 5371.7×-1728.8	0.9997	0.55	3.45
E1-5OH-PCB149	Y = 6502.2×-2647.4	0.9998	0.53	0.99
E2-5OH-PCB149	Y = 4969.6×-624.2	0.9999	0.60	3.37

^a^, All standard concentration, 10.0 ng mL^-1^.

### Determination of chiral OH-PCBs in PB induced rat liver microsomal samples

**Figure 4:**
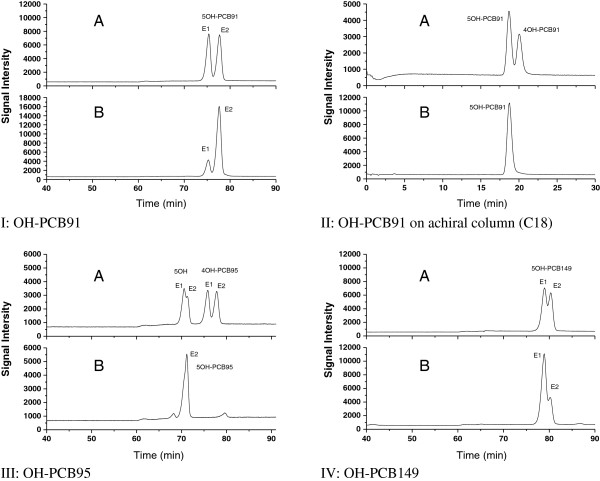
Chromatograms of OH-PCBs formed in in rat liver microsomal incubations (B) and their relevant standards (A).

Though PCBs are a group of persistent organic pollutants, many PCB congenrs can be metabolized by a series of enzymes in biota. Hydroxylated metabolites of PCBs are a group of important metabolites and have been frequently reported in biota [28,29,34]. Rat liver microsomes are a powerful tool to study the metabolism of PCBs and generate atropisomerically enriched OH-PCBs [32]. The newly developed HPLC-MS method was applied to the determination of the atropisomers of the hydroxylated metabolites of chiral PCB91, PCB95 and PCB149 formed by liver microsomes from PB-treated rats. Furthermore, a large amount of these chiral OH-PCBs were produced from chiral PCBs by PB induced rat liver microsomes, reaching μg mL^-1^ level. It can be seen from Table 3 that good spiked recoveries (%) of 4OH-PCB159 in these samples ranged from 82.3 ± 4.3 to 96.5 ± 9.0 in LC-MS method. In addition, 5OH-PCB91, 95 and 149 were detected with high concentrations and no 4OH-PCB 91, 95 and 149 were found, which suggested that liver microsomes for PB-pretreated rats preferentially produce para-position hydroxylated metabolites of these chiral PCBs. More interestingly, these chiral 5OH-PCBs were formed enantioselectively. It can be seen from Figure 4 and Table 3 that the second eluent (E2) of 5OH-PCB91 and the first eluent (E1) of 5OH-PCB149 were enriched, with EFs being 0.183 ± 0.002 for 5OH-PCB91, and 0.731 ± 0.007 for 5OH-PCB149. However, only E2-OH-PCB95 was found in HPLC-MS method.

**Table 3:** Comparison of OH-PCBs atropisomer levels and enantiomeric fractions (EF) determined by LC-MS and GC-ECD methods

**Sample**	**Congener**	**LC-MS**	**GC-ECD**	**GC-ECD**
		**Nucleodex β-PM**	**BDM chiral column**	**DB1 achiral column**
PCB91	E1-5OH-PCB91 (μg mL^-1^)	0.306 ± 0.011	0.268 ± 0.006	1.334 ± 0.023
	E2-5OH-PCB91 (μg mL^-1^)	1.364 ± 0.068	1.160 ± 0.038	
	Total (μg mL^-1^)	1.670 ± 0.079	1.428 ± 0.044	
	EF (5OH-PCB91)	0.183 ± 0.002	0.188 ± 0.001	
	4OH-PCB91	ND	ND	0.013 ± 0.001
	Recovery of 4OH-PCB159 (%)	82.3 ± 4.3	97.3 ± 1.7	99.9 ± 3.6
PCB95	E1-5OH-PCB95 (μg mL^-1^)	ND	0.262 ± 0.014	1.183 ± 0.068
	E2-5OH-PCB95 (μg mL^-1^)	1.346 ± 0.345	0.789 ± 0.042	
	Total (μg mL^-1^)	1.346 ± 0.345	1.051 ± 0.057	
	EF (5OH-PCB95)		0.249 ± 0.001	
	4OH-PCB95	ND	ND	0.007 ± 0.002
	Recovery of 4OH-PCB159 (%)	96.5 ± 9.0	106.6 ± 11.4	101.5 ± 9.1
PCB149	E1-5OH-PCB149 (μg mL^-1^)	0.913 ± 0.079	0.648 ± 0.019	1.281 ± 0.050
	E2-5OH-PCB149 (μg mL^-1^)	0.335 ± 0.029	0.272 ± 0.009	
	Total (μg mL^-1^)	1.248 ± 0.108	0.920 ± 0.028	
	EF(5OH-PCB149)	0.731 ± 0.007	0.704 ± 0.003	
	Recovery of 4OH-PCB159 (%)	88.6 ± 0.7	113.8 ± 1.9	115.4 ± 1.2
Blank control	Any OH-PCBs	ND	ND	ND
	Recovery of 4OH-PCB159 (%)	92.5	-	111

ND: not detected.

In order to confirm the validity of LC-MS method for the atropisomers of these chiral OH-PCBs, the samples were also analyzed by GC-ECD method with derivatization on chiral and achiral columns. Results showed that the EFs and each mass of the atropisomers were highly consistent in LC and GC methods. For example, EFs were 0.188 ± 0.001 for 5OH-PCB 91 and 0.704 ± 0.003 for 5OH-PCB 149 for the GC method and EFs were 0.183 ± 0.002 for 5OH-PCB 91 and 0.731 ± 0.007 for 5OH-PCB 149 for LC method, respectively. In addition, the total mass of OH-PCBs on a GC chiral and achiral column also matched well the total mass on LC-MS column. The total mass of 5OH-PCB91 was 1.670 ± 0.079 μg mL^-1^ for HPLC-MS method; the total masses of 5OH-PCB91 were 1.428 ± 0.044 μg mL^-1^ and 1.334 ± 0.023 μg mL^-1^ on chiral and achiral column for GC-ECD method. However, 5OH-PCB 95 showed the difference because chiral LC column cannot separate two atropisomers of 5OH-PCB 95 well and the atropisomer of lower concentration (E1) is off-set by the E2 with higher concentration. Therefore, chiral HPLC columns with improved resolution for chiral OH-PCBs should be the subject of research in the future. In addition, a trace amount of 4OH-PCB91 and 4OH-PCB95 (less than 1% mass ratio of their 5OH-PCB91 and 5OH-PCB95) were found on achiral column of GC-ECD method but not on chiral LC and GC methods.

### Experimental

#### Materials

**Figure 5:**
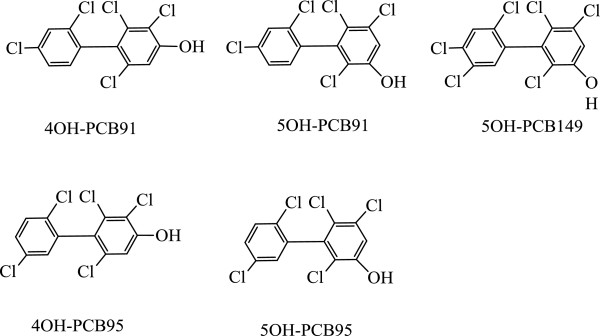
Structures of five chiral OH-PCBs.

The chemical structures of five chiral OH-PCBs (4OH-PCB91, 5OH-PCB91, 4OH-PCB95, 5OH-PCB95 and 5OH-PCB149; 98% purity or better) are shown in Figure 5. They were synthesized by the Synthesis Core of the Iowa Superfund Research Program at the University of Iowa using established procedures [35,36]. The recovery standard, 4OH-PCB159 from AccuStandard was used for LC and GC methods. The internal standards, PCB166 and PCB204, for GC analyses were obtained from AccuStandard (New Haven, CT, USA). Stock solutions of these OH-PCBs (1.0 mg mL^-1^) were prepared in acetonitrile. Working solutions of these OH-PCBs were prepared by gradual dilution of the stock solution with acetonitrile. All standards and solutions of these OH-PCBs were stored hermetically in amber glass vials at 4°C.

Acetonitrile was HPLC grade and hexane, 2-propanol and methyl tert-butyl ether (MTBE) were pesticide grade solvents purchased from Fisher Scientific (Pittsburgh, PA). The deionized water (18.2 MΩ) was from an ultrapure water system (Barnstead International, Dubuque, IA, USA). All other chemicals and reagents used in this experiment were of analytical reagent grade or better.

## LC-MS method

Qualitative and quantitative analysis of chiral OH-PCBs was performed on HPLC-MS (Agilent 1100 Series LC/MSD) with an autosampler. Sample separation was optimized using Nucleodex β-PM (4 mm × 200 mm, 5 μm) and Nucleodex β-OH (4 mm × 200 mm, 5 μm) from Macherey-Nagel, Germany; Astec Chirobiotic V (4.6 mm × 250 mm, 5 μm) from Sigma-Aldrich; LichroCART 250–4 ChiraDex (4 mm × 250 mm, 5 μm) from Merck; Agilent SB-C18 (2.1 × 50 mm, 1.8 μm). The mobile phase was acetonitrile and water with isocratic conditions. The injection volume of each column was 20 μl except for Agilent SB-C18 (5 μl). MS electrospray in negative ionization mode (LC-ESI (─)-MS) was utilized. Other operating conditions were: Selected Ion Monitoring (SIM) Ion Mass: *m/z* 341 for OH-PCB91 and OH-PCB95; *m/z* 375 for OH-PCB149 and OH-PCB159; Fragmentor: 100 V; Capillary Voltage: 4000 V; Gain: 7.00; Drying Gas Flow: 10 L min^-1^; Nebulizer Pressure: 35 psi; Drying Gas Temperature: 250°C.

### GC-ECD methods

Analysis of the methylated derivatives of OH-PCBs was carried out using an Agilent 6890 N gas chromatograph equipped with a ^63^Ni-μECD detector and a DB1-MS capillary column (60 m × 0.25 mm ID × 0.25 μm film thickness; Agilent, Santa Clara, CA, USA) [32]. The injector and detector temperatures were 280°C and 300°C, respectively. The temperature program was as follows: 100°C for 1 min, 5°C/min to 250°C, hold for 20 min, 5°C/min to 280°C, hold for 3 min. Helium was used as the carrier gas with a flow rate of 1 mL/min. Concentrations of OH-PCB metabolites were determined using PCB 166 or PCB 204 as internal standards. The detection limits (LODs) of the OH-PCBs, calculated from the standard deviation (SD) of the response and the slope of the calibration curve (S) using the formula LOD = 3.3(SD/S) [37], were 22.1 ng mL^-1^, 23.0 ng mL^-1^, 19.0 ng mL^-1^, 27.6 ng mL^-1^ and 26.6 ng mL^-1^ for 5OH-PCB 91, 4OH-PCB 91, 5OH-PCB 95, 4OH-PCB 95 and 5OH-PCB 149, respectively.

Enantioselective analysis was performed using an Agilent 7890A gas chromatograph equipped with ^63^Ni μECD detector as described previously [31]. The injector and detector temperatures were kept at 250°C. The atropisomers of five chiral OH-PCBs were separated using a ChiralDex BDM column (30 m × 250 μm × 0.12 μm; Supelco, Analytical, St. Louis, MO). The temperature program was as follows: 100°C for 1 min, 10°C/min to 148°C, hold for 400 min, 10°C/min to 200°C, hold for 13 min. The flow rate of the carrier gas (helium) was 3 mL/min.

### Calculation of enantiomeric fractions

Enantiomer fraction or enantiomeric fraction (EF) was used to calculate the atropisomer composition in this work: EF = C_E1_/( C_E1_ + C_E2_).

where C_E1_ and C_E2_ are the concentrations (C) of the first-eluting enantiomer (E1) and the second-eluting enantiomer (E2) on the chiral chromatographic column.

### Microsomal metabolism experiments

Experiments involving animals were approved by the Institutional Animal Care and Use Committee at the University of Iowa. Liver microsomes were prepared from phenobarbital-pretreated male Spargue-Dawley rats and microsomal metabolism experiments were performed as described elsewhere [32]. Briefly, an incubation mixture (16 mL final volume) consisting of phosphate buffer (0.1 M, pH 7.4), nicotinamide adenine dinucleotide phosphate hydrogen (NADPH) (0.5 mM), magnesium chloride (3 mM), and hepatic microsomal protein (0.75 mg/mL) was pre-incubated for 5 min at 37 ± 1°C in a shaking water bath. PCB 91, PCB95 or PCB149 were added to initiate the reactions, with a final concentration at 50 μM. Ten mL of ice cold sodium hydroxide (0.5 M) were added to stop the reaction after 30 min incubation. Control incubations without PCBs were performed in parallel. Extraction of each PCB and its hydroxylated metabolites was performed using a published method [32]. Surrogate standard (1370 ng of 4OH-PCB 159) was added to each sample, followed by hydrochloric acid (6 M, 5 mL) and 2-propanol (15 mL). The samples were extracted with hexane-MTBE (1:1 v/v, 25 mL) and hexane (15 mL). The combined organic extracts were washed with an aqueous KCl solution (1%, 15 mL). After removal of the organic phase, the KCl phase was re-extracted with hexane (15 mL), and the combined extracts were reduced under a gentle stream of nitrogen to dryness. The residue was redissolved in 3 mL of hexane and divided by weight into two parts for LC-MS and GC-ECD analysis. The samples were blown to dryness under a gentle stream of nitrogen and then redissolved in acetonitrile for LC-MS analysis. For GC analysis, the hydroxylated metabolites were derivatized with diazomethane and subjected to a sulfur cleanup step prior to analysis as described previously [25].

## Conclusions

A novel HPLC-MS method was developed and successfully applied to measure chiral OH-PCB levels and EFs in rat liver microsomal samples. The method can easily detect chiral OH-PCBs without derivatization. The results of OH-PCB atropisomers from LC-MS were consistent with those from GC-ECD method, which confirm the validity of LC-MS method for the determination of OH-PCB atropisomers. It is the first report of chiral OH-PCBs detected by HPLC-MS. The proposed method should be applicable to detect chiral OH-PCBs in environmental samples and metabolic mechanisms of PCBs as well as rat microsomes.

## Competing interests

The authors declare that they have no competing interests.

## Authors’ contributions

GZ and XW performed the experiments and drafted the manuscript. HL and JS participated in the conception, design of study and revision of the manuscript. All authors read and approved the final manuscript.
